# Novel Insights into Normal Karyotype Miscarriage: Distinctive Patterns and Implications for Diagnosis Based on Next‐Generation Sequencing Analysis

**DOI:** 10.1002/rmb2.70053

**Published:** 2026-05-11

**Authors:** Yumene Kubota, Tomoko Ichikawa, Shigeru Matsuda, Yoshimitsu Kuwabara, Shunji Suzuki, Yasuyuki Negishi

**Affiliations:** ^1^ Department of Obstetrics and Gynecology Nippon Medical School Tokyo Japan; ^2^ Department of Microbiology and Immunology Nippon Medical School Tokyo Japan

**Keywords:** biochemical pregnancy loss, chromosomal analysis of chorionic villus, early miscarriage, normal karyotype miscarriage, subchorionic hematoma

## Abstract

**Purpose:**

This study aimed to identify the clinical differences between normal and abnormal karyotype miscarriages and evaluate whether clinical findings could predict the presence or absence of chromosomal abnormalities.

**Methods:**

A total of 117 patients with early miscarriage who underwent chorionic villus chromosomal testing using next‐generation sequencing were retrospectively analyzed. Clinical findings were compared between the normal (euploid; *n* = 47) and abnormal karyotype (aneuploid; *n* = 70) groups.

**Results:**

Normal karyotype miscarriages occurred significantly more often before yolk sac visualization (29.8% vs. 0%, *p* < 0.0001) and before fetal heartbeat confirmation (51.1% vs. 15.7%, *p* = 0.0001) than abnormal karyotype miscarriages. The mean crown–rump length was larger in the normal karyotype group (15.3 ± 8.2 mm vs. 11.8 ± 6.8 mm, *p* = 0.037). Normal karyotype miscarriages exhibited a bimodal distribution in gestational age at miscarriage. In addition, the normal karyotype was associated with a history of two or more biochemical pregnancy losses (21.3% vs. 7.1%, *p* = 0.045).

**Conclusion:**

Distinct clinical and ultrasonographic patterns were observed in normal and abnormal karyotype miscarriages. These features have limited diagnostic value, and their usefulness in supporting counseling or decision‐making is also limited when genetic testing is not available.

## 1 Introduction

Miscarriages occur in approximately 10%–15% of all pregnancies [1] with 40%–60% of early miscarriages attributable to embryonic chromosomal abnormalities in recurrent pregnancy loss (RPL) [2]. Chromosomal analysis of pregnancy products of conception (POC) provides crucial information for determining whether a miscarriage is associated with chromosomal abnormalities and serves as a guideline for deciding subsequent pregnancy management strategies. In general, if embryonic abnormalities are identified in a case of sporadic miscarriage, no specific therapeutic intervention is required for subsequent pregnancies. However, in RPL cases where embryonic aneuploidy is repeatedly suspected, preimplantation genetic testing for aneuploidy (PGT‐A) may be considered an option after appropriate counseling. Conversely, when the karyotype is normal, particularly in cases of RPL, additional investigations are required to identify the underlying cause. Maternal factors to be considered include antiphospholipid antibodies, uterine malformations, metabolic diseases, thyroid dysfunction, abnormalities of the cervical or endometrial microbiota, and obesity, all of which are supported by more recent studies [3, 4, 5, 6]. More recently, associations between the uterine microbiota and RPL have also been reported [7]. These findings emphasize the multifactorial nature of RPL and the need for comprehensive maternal assessment. Furthermore, effective treatments have been reported for several underlying causes, such as anticoagulant therapy with heparin or aspirin for antiphospholipid syndrome [8].

Traditionally, G‐banding karyotyping has been used to analyze POC. However, this method has several limitations, including false negative results owing to maternal cell contamination, culture failure, and the inability to obtain samples when the products are expelled at home [9]. Recently, next‐generation sequencing (NGS) has been introduced for the chromosomal analysis of miscarriage tissues. As it does not require cell culture, NGS minimizes the risk of maternal cell contamination [10] and allows for analysis even in specimens collected after spontaneous expulsion. Nonetheless, NGS is not universally available, as it is generally offered as a self‐paid test and is only available at limited facilities.

Moreover, in cases of complete miscarriage, chorionic tissue may not be retrievable and thus cannot be submitted for testing. Estimation of the presence of chromosomal abnormalities in miscarriages based on clinical findings has significant clinical implications. This study aimed to identify the clinical differences between normal and abnormal karyotype miscarriages and evaluate whether clinical findings, particularly transvaginal ultrasound features, could predict the presence or absence of chromosomal abnormalities. In a prespecified exploratory subset, we also examined endometrial microbiota obtained in the mid‐luteal phase of the second or third menstrual cycle after the miscarriage to generate hypotheses regarding euploid miscarriages.

The novelty of this study lies in its focus on NGS‐confirmed euploid early miscarriages. While most previous studies have relied on G‐banding analysis, our study employed chromosomal testing of chorionic villi using NGS in all cases, enabling a more accurate evaluation of the clinical and ultrasonographic characteristics of euploid miscarriages.

## 2 Materials and Methods

### 2.1 Study Population

**FIGURE 1:**
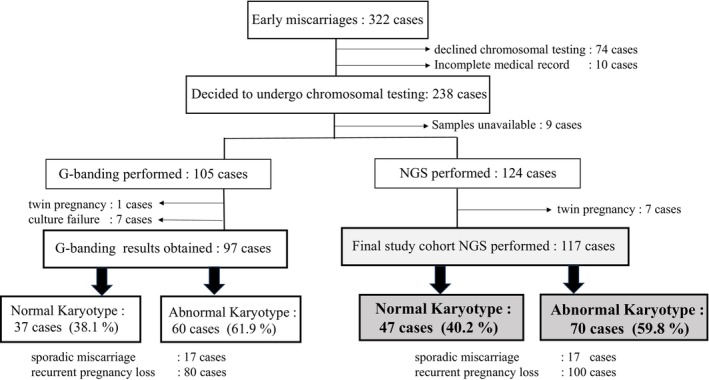
Flowchart of patient selection. Flowchart illustrating patient selection and the chromosomal testing process. Among 322 patients diagnosed with early miscarriage between January 1, 2021, and May 31, 2025, 238 decided to undergo chorionic villus chromosomal testing. After excluding nine patients who were unable to attend the hospital or submit villous samples, chromosomal testing was attempted in 229 patients. Among these patients, 105 selected G‐banding analysis; after excluding one twin pregnancy and seven cases with culture failure, evaluable G‐banding results were obtained for 97 patients. Separately, 124 patients selected next‐generation sequencing (NGS)–based analysis; after excluding seven twin pregnancies, 117 patients underwent NGS testing, all of whom yielded interpretable chromosomal results. Because the present study focused on the clinical characteristics of miscarriage based on NGS‐derived chromosomal results, only the 117 patients who underwent NGS‐based analysis were included in the final analytic cohort. Detailed reasons for exclusion are shown in the figure.

This retrospective observational study included patients who experienced early pregnancy loss before 12 gestational weeks and underwent chromosomal analysis of products of conception (POC) at our institution between January 1, 2021, and May 31, 2025. The overall study design and patient selection process are summarized in Figure 1.

At our institution, all patients diagnosed with early miscarriage are routinely provided with information regarding chorionic villus chromosomal testing. While chromosomal testing is primarily recommended for patients with RPL, it is also offered to patients who experience a miscarriage for the first‐time upon request and after appropriate counseling.

During counseling, both G‐banding and NGS‐based chromosomal analyses are explained in detail, including their methodological characteristics, limitations, and financial aspects. Patients are informed that G‐banding analysis for first‐time miscarriages is self‐funded and that for second or subsequent miscarriages has been covered by the Japanese national health insurance system since April 2022, whereas NGS‐based analysis is self‐funded irrespective of miscarriages status.

Written informed consent was obtained from all patients who underwent self‐funded chromosomal testing. For NGS‐based chromosomal analysis, additional written consent approved by the institutional ethics committee was obtained.

Patients who decided to undergo chromosomal testing after counseling were included in the study. Clinical characteristics were retrospectively analyzed according to the NGS‐derived chromosomal results.

The following clinical parameters were analyzed: rate of normal karyotype, maternal and paternal age, parity, history of miscarriage, history of biochemical pregnancy loss (BPL), mode of conception, presence of risk factors for RPL and its treatments, presence of yolk sac (YS), fetal heartbeat (FHB), gestational sac, crown‐rump length (CRL), presence of subchorionic hematoma (SCH), and whether specimens were obtained through natural expulsion or surgical intervention. The clinical characteristics of the study participants are presented in Table 1.

**TABLE 1:** Comparison of participant characteristics between normal and abnormal karyotype groups for early miscarriage.

Variable/Category	Euploid (*n* = 47)	Aneuploid (*n* = 70)	*p*
Patient characteristics			
Maternal age, median^a^	35.0 (24.0–45.0)	38.0 (27.0–46.0)	0.014^b^
Paternal age, median^a^	37.0 (27.0–59.0)	38.5 (26.0–51.0)	0.351^b^
Parity, median^a^	0.00 (0.00–1.00)	0.00 (0.00–2.00)	0.018^c^
Number of previous miscarriages, *n* (%)			
0	8 (17.0)	9 (12.9)	0.356^c^
1	14 (29.8)	20 (28.6)	0.523^c^
≥ 2	25 (53.1)	41 (58.6)	0.453^c^
Number of previous biochemical miscarriages, *n* (%)			
0	29 (61.7)	53 (75.7)	0.078^c^
1	8 (17.0)	12 (17.1)	0.596^c^
≥ 2	10 (21.3)	5 (7.1)	0.045^c^
Mode of conception, *n* (%)			
Natural conception	16 (34.0)	24 (34.3)	0.569^c^
Timed intercourse	2 (4.2)	6 (8.6)	0.304^c^
IUI	0 (0)	3 (4.3)	0.210^c^
ART	29 (61.7)	37 (52.9)	0.225^c^
Treatment, *n* (%)			
No treatment	19 (40.4)	25 (35.7)	0.373^c^
LDA	17 (36.2)	33 (47.1)	0.162^c^
HA	11 (23.4)	12 (17.1)	0.273^c^
Mode of villus collection, *n* (%)			
Natural expulsion	18 (38.3)	21 (30.0)	0.231^c^
Surgical intervention	29 (61.7)	49 (70.0)	0.231^c^

*Note:* ART includes in vitro fertilization (IVF) and intracytoplasmic sperm injection (ICSI). “Number of previous miscarriages” refers exclusively to clinical miscarriages. Biochemical pregnancy losses are reported separately as “Number of previous biochemical miscarriages.” Natural expulsion indicates spontaneous expulsion of villi without surgical management. Surgical intervention indicates inpatient miscarriage management using a manual vacuum aspiration kit.

Abbreviations: HA, heparin plus aspirin therapy; IUI, intrauterine insemination; LDA, low‐dose aspirin therapy; n.s., not significant.

^a^ Data are presented as median (interquartile).

^b^ Data are compared using the Mann–Whitney test.

^c^ Data are compared using Fisher's exact test.

In the present analysis, “previous miscarriages” refers exclusively to clinically recognized miscarriages confirmed by ultrasonography. BPLs were excluded from this definition; hence, BPL data were recorded and analyzed separately.

Villus samples were obtained either through natural expulsion or surgical intervention. Surgical intervention was defined as inpatient miscarriage management using a manual vacuum aspiration kit, whereas natural expulsion referred to spontaneous passage of villi without surgical management.

Written informed consent was obtained from all patients who elected to undergo NGS testing. The collection and use of miscarriage specimens were approved by the Ethics Committee of the Nippon Medical School Hospital (A‐2021‐058). All procedures were performed in accordance with the institutional regulations and ethical principles of the Declaration of Helsinki.

### 2.2 Definition of RPL and Assessment of Maternal Risk Factors

Among the 47 women who underwent RPL screening, 39 were classified as RPL, defined as two or more pregnancy losses, whereas eight were classified as non‐RPL due to being tested after their first clinical miscarriage. Patients in the non‐RPL group were also screened for RPL because they had a history of recurrent BPLs, which are considered within the spectrum of RPL according to the ESHRE guideline.

For the analysis of maternal risk factors in euploid miscarriages, the number and percentage of patients with each risk factor were calculated using the total number of euploid miscarriage cases (*n* = 47) as the denominator. Because individual patients could have more than one risk factor, overlapping cases were allowed, and therefore, the sum of percentages exceeds 100%.

Patients who experienced two or more miscarriages underwent a comprehensive evaluation for RPL at our institution. This evaluation included transvaginal ultrasonography to assess uterine anomalies, blood tests for thrombophilia factors, and parental chromosomal analysis when requested. In addition, even after a first clinical miscarriage, RPL evaluation was occasionally performed in patients with a history suggestive of reproductive failure, such as recurrent BPL or repeated implantation failure, particularly upon persistent patient request.

Patients with uterine malformations or parental chromosomal abnormalities were not excluded from the study, as these conditions represent potential maternal or parental risk factors associated with miscarriage. These factors were included in the analysis of RPL‐related risk factors in the normal karyotype group.

### 2.3 Determination of Gestational Age

While confirming the exact date of miscarriage would provide valuable information, the number of days from miscarriage to diagnosis varies substantially between cases. Therefore, in this study, 117 cases of early miscarriages were classified according to karyotype (normal vs. abnormal) using an ultrasonography‐based grouping method: (1) miscarriages occurring before FHB confirmation and (2) those in this category further subdivided into five groups based on CRL, which is internationally recognized as the standard for first‐trimester dating [11]. The Robinson and Fleming formula for CRL is consistent with recommendations from major guidelines, including those of the American College of Obstetricians and Gynecologists [12], and the Japan Society of Obstetrics and Gynecology [13], all of which state that CRL is the most accurate method for gestational age determination in early pregnancy. The following groups were defined: (i) before FHB confirmation, (ii) CRL < 10 mm, (iii) 10 mm ≤ CRL < 16 mm (corresponding to 7 weeks of gestation), (iv) 16 mm ≤ CRL < 24 mm (approximately 8 weeks), (v) 24 mm ≤ CRL < 30 mm (approximately 9 weeks), and (vi) 30 mm ≤ CRL < 41 mm (approximately 10 weeks).

### 2.4 Chromosomal Analysis of POC Using NGS

Chromosomal abnormalities in POC were analyzed using NGS. Miscarriage tissues were obtained either after spontaneous expulsion or during surgical evacuation. Villous tissue was carefully separated from the maternal decidua to minimize maternal cell contamination. The samples were washed, trimmed into approximately 1 cm fragments, placed in sealed containers, and stored at ≤ −18°C until analysis. Genomic DNA was extracted and subjected to NGS‐based chromosomal copy number variation analysis. All procedures, including DNA extraction, library preparation, sequencing, and bioinformatic analysis, were performed by Ovus Co. Ltd. (Tokyo, Japan) using a commercially available platform.

### 2.5 Antithrombotic Treatment Strategy

In the normal karyotype group, 25 patients (53%) had a history of two or more pregnancy losses. Additionally, some patients with only one previous miscarriage with thrombophilia received low‐dose aspirin (LDA) at 100 mg/day alone or in combination with unfractionated heparin (heparin calcium, 10,000 units/day). The indications for antithrombotic therapy included a history of late miscarriage, intrauterine fetal death, preeclampsia, or a prior miscarriage with confirmed euploid embryonic karyotype. These treatment indications were predefined and approved by the Ethics Committee.

All patients diagnosed with antiphospholipid syndrome (APS) received standard antithrombotic therapy consisting of LDA and unfractionated heparin. LDA was initiated either after the elevation of basal body temperature in natural conception cycles or on the day following embryo transfer in assisted reproductive technology cycles. Unfractionated heparin was started after confirmation of pregnancy and continued until miscarriage diagnosis.

### 2.6 Endometrial Microbiota Analysis

The endometrial microbiota was analyzed using a commercial endometrial flora test kit (Varinos, Tokyo, Japan). NGS was based on 16S rRNA gene sequencing. Endometrial samples (*n* = 18) were obtained in the mid‐luteal phase (approximately 7 days after ovulation) of the second or third menstrual cycle after the miscarriage. These samples were collected with utmost care to minimize contamination, and DNA was extracted and subjected to NGS‐based microbiota analysis. DNA extraction and sequencing were outsourced to another provider (Varinos). In this study, we defined a normal endometrial microbiota as a *Lactobacillus*‐dominant microbiota (LDM), with *Lactobacillus* comprising ≥ 90% of the total microbiota. Samples with < 90% *Lactobacillus* were classified as non‐*Lactobacillus*‐dominant microbiota (NLDM), indicating abnormal endometrial microbiota.

### 2.7 Statistical Analysis

Statistical analyses were performed using the GraphPad Prism software (GraphPad Software, San Diego, CA, USA). Continuous variables were expressed as the mean ± standard deviation (SD) and compared using the Mann–Whitney *U* test. Categorical variables were compared using Fisher's exact test, and a *p* value < 0.05 (two‐tailed) was considered significant.

## 3 Results

### 3.1 Patient Selection and Study Population

Between January 1, 2021, and May 31, 2025, a total of 322 patients diagnosed with early miscarriage at our institution received counseling regarding chorionic villus chromosomal testing (Figure 1). Among them, 74 declined chromosomal testing, and 10 were excluded owing to incomplete medical records. The remaining 238 patients decided to undergo chromosomal testing. Of these, nine were unable to attend the hospital or submit villous samples because of intercurrent conditions, such as fever, resulting in 229 patients for whom chromosomal testing was attempted. Among these patients, 105 selected G‐banding analysis. After excluding one twin pregnancy and seven cases with culture failure, evaluable G‐banding results were obtained for 97 patients. Among the 124 patients who selected NGS‐based chromosomal analysis, seven with twin pregnancies were excluded, leaving 117 patients who underwent NGS testing. Interpretable chromosomal results were successfully obtained in all 117 NGS cases, with no exclusions due to test failure.

Because the present study was specifically designed to evaluate the clinical characteristics of early miscarriage based on NGS‐derived chromosomal results, cases analyzed using G‐banding were excluded from further analysis. Accordingly, the final study population consisted of 117 patients who underwent NGS‐based chromosomal analysis, regardless of whether the miscarriage represented a first or recurrent event. Based on the NGS results, 47 cases (40.2%) were classified as euploid, and 70 cases (59.8%) as aneuploid. Subsequent analyses focused on comparing the clinical characteristics between these two groups.

### 3.2 Comparison of Baseline Characteristics Between the Normal and Abnormal Karyotype Groups

Among the 117 patients analyzed, 47 (40.2%) and 70 (59.8%) had normal and abnormal karyotypes, respectively (Table 1). The maternal age was significantly higher in the abnormal karyotype group than in the normal karyotype group (median [IQR]: euploid 35.0 [24.0–45.0] vs. aneuploid 38.0 [27.0–46.0]; *p* = 0.014). In contrast, the paternal age did not differ significantly between the two groups (median [IQR]: euploid 37.0 [27.0–59.0] vs. aneuploid 38.5 [26.0–51.0]; *p* = 0.351). The number of previous miscarriages was comparable between the two groups. However, two or more BPLs were significantly more frequent in the normal karyotype group than in the abnormal karyotype group (10/47 [21.3%] vs. 5/70 [7.1%], *p* = 0.045; Table 1). Parity was significantly lower in the normal karyotype group (median 0 [0–1]) than in the abnormal karyotype group (median 0 [0–2], *p* = 0.018; Table 1). No significant differences were observed in the mode of conception (natural conception, timed intercourse, IUI, or assisted reproductive technology (ART)) between the two groups (Table 1). In the normal karyotype group, villi were obtained by natural expulsion in 18 cases (38.3%) and by surgical intervention in 29 cases (61.7%). In the abnormal karyotype group, 21 patients (30.0%) underwent natural expulsion and 49 (70.0%) underwent surgical intervention. There was no significant difference in the mode of villus collection between the two groups (*p* = 0.231).

### 3.3 Risk Factors for RPL in the Normal Karyotype Group

Among maternal risk factors identified in the normal karyotype group, APS was observed in three cases (6.3%), and transient antiphospholipid antibody positivity in 10 cases (21.2%). Other abnormalities included uterine malformations in three cases (6.3%), parental chromosomal abnormalities in two cases (4.2%), and thyroid dysfunction in nine cases (19.1%). No risk factors were identified in 23 cases (48.9%).

Among the 47 cases with normal karyotypes, several patients were found to have potential risk factors for RPL based on the evaluation results. Uterine malformations were identified in three cases, and parental chromosomal abnormalities were identified in two cases. In addition, some patients had multiple coexisting RPL risk factors.

### 3.4 Treatments for RPL in the Normal and Abnormal Karyotype Groups

For RPL treatment, patients received either LDA at 100 mg/day, heparin calcium 10,000 units/day (alone or in combination with LDA), or no treatment. There were no significant differences in the treatment modalities between the normal and abnormal karyotype groups (Table 1).

### 3.5 Normal Karyotype Miscarriages Typically Occur Before the FHB or YS Detection

Among the 47 cases with normal karyotypes, 24 (51.1%) occurred before FHB confirmation, which was significantly more frequent than in the abnormal karyotype group (euploid: 24/47 [51.1%] vs. aneuploid: 11/70 [15.6%], *p* = 0.0001; Table 2). Miscarriages occurring before YS confirmation were observed in 14 cases, all of which belonged to the normal karyotype group, demonstrating a statistically significant difference (euploid: 14/47 [29.8%] vs. aneuploid: 0/70 [0%], *p* < 0.0001; Table 2). SCH was detected in 11 of 117 cases, with a significantly higher incidence in the normal karyotype group (euploid: 8/47 [17.0%] vs. aneuploid: 3/70 [4.2%], *p* = 0.0241; Table 2). Among the miscarriages that occurred after FHB confirmation, the CRL was significantly larger in the normal karyotype group than in the abnormal karyotype group (euploid: 15.3 ± 8.2 mm vs. aneuploid: 11.8 ± 6.8 mm, *p* = 0.037; Table 2).

**TABLE 2:** Comparison of clinical findings between normal and abnormal karyotype groups for early miscarriage.

Clinical finding, *n* (%)	Euploid (*n* = 47)	Aneuploid (*n* = 70)	*p*
Miscarriage before FHB confirmation	24 (51.1)	11 (15.7)	0.0001^a^
Miscarriage before YS confirmation	14 (29.8)	0 (0)	< 0.0001^a^
The presence of SCH	8 (17.0)	3 (4.2)	0.0241^a^
CRL at miscarriage after FHB confirmation (mean ± SD, mm)	15.3 ± 8.2	11.8 ± 6.8	0.037^b^

Abbreviations: CRL, crown–rump length; FHB, fetal heartbeat; SCH, subchorionic hematoma; YS, yolk sac.

^a^ Data are compared using Fisher's exact test.

^b^ Data are compared using the Mann–Whitney test.

### 3.6 Distinct Temporal Patterns of Miscarriage by Karyotype Status

**FIGURE 2:**
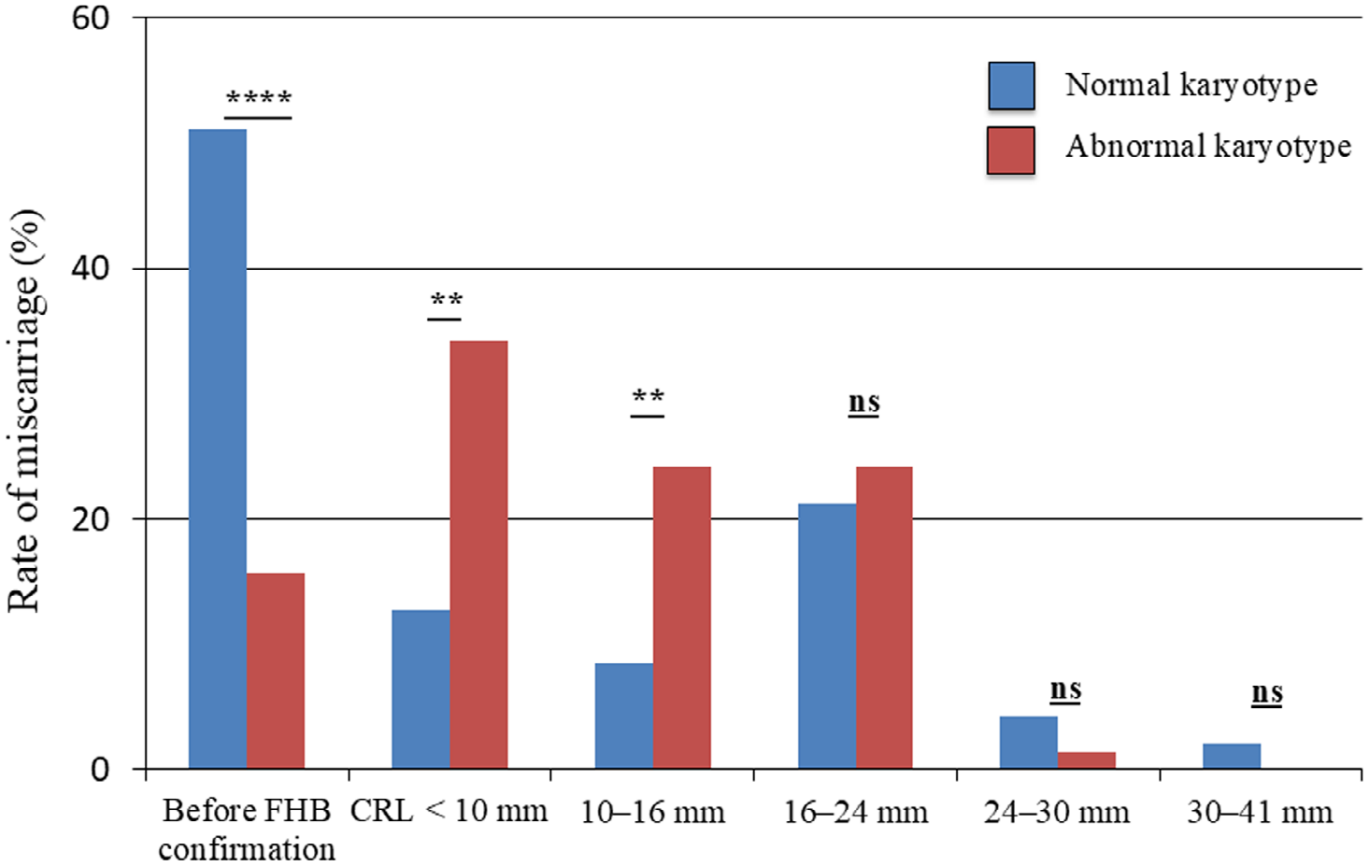
Distribution of miscarriage timing according to karyotype classification. The proportion of miscarriages with normal and abnormal karyotypes is shown across six categories based on ultrasound findings: (i) before fetal heartbeat (FHB) confirmation, (ii) CRL < 10 mm, (iii) 10–16 mm (corresponding to ~7 weeks of gestation), (iv) 16–24 mm (approximately 8 weeks), (v) 24–30 mm (~9 weeks), and (vi) 30–41 mm (~10 weeks). Normal karyotype miscarriages showed two peaks, occurring most frequently before FHB confirmation and again at approximately 8 weeks, whereas abnormal karyotype miscarriages exhibited a single peak when the CRL was < 10 mm. Statistical significance was calculated using Fisher's exact test, with **p < 0.01, ****p < 0.0001, and ns (not significant) indicated.

The temporal distribution of miscarriages in the normal and abnormal karyotype groups is shown in Figure 1. Normal karyotype miscarriages were predominantly observed before FHB confirmation, whereas abnormal karyotype miscarriages were uncommon prior to FHB confirmation but occurred more frequently thereafter. Within the CRL range of > 10 to ≤ 16 mm, miscarriages with abnormal karyotypes were more frequently observed. When the CRL exceeded 16 mm, no significant difference was observed between normal‐ and abnormal‐karyotype miscarriages (Figure 2).

### 3.7 Endometrial Dysbiosis Is Associated With Normal Karyotype Miscarriages Before FHB and YS Confirmation

Exploratory subset analysis was performed to further identify the factors associated with normal karyotype miscarriage. Among the 47 normal karyotype miscarriages, endometrial microbiota analysis was performed in 18 cases at the patient's request. Of these, 11 cases exhibited LDM, whereas seven showed NLDM (Table 3). Although the difference was not statistically significant after FHB confirmation (*p* = 0.056), six of the seven NLDM miscarriages occurred before FHB confirmation compared with four of the 11 LDM miscarriages, and six of the seven NLDM miscarriages occurred before YS confirmation compared with two of the 11 LDM miscarriages (*p* = 0.009). These findings suggest that endometrial dysbiosis may be strongly associated with normal karyotype miscarriages occurring before FHB and YS confirmation.

**TABLE 3:** Association between endometrial microbiota status and miscarriage timing.

Clinical finding, *n* (%)	LDM (*n* = 11)	NLDM (*n* = 7)	*p* ^a^
Miscarriage before FHB confirmation, YS—	2 (18.2)	6 (85.7)	0.009
Miscarriage before FHB confirmation, YS+	2 (18.2)	0 (0)	0.359
Miscarriage after FHB confirmation	7 (63.6)	1 (14.3)	0.056

Abbreviations: FHB, fetal heartbeat; LDM, *Lactobacillus*‐dominant microbiota; NLDM, non‐*Lactobacillus*‐dominant microbiota; YS, yolk sac.

^a^ Data are compared using Fisher's exact test.

## 4 Discussion

To date, many studies have examined the relationship between ultrasonographic findings and embryonic chromosomal abnormalities in early miscarriage; however, most relied on conventional G‐banding analysis, which is limited in accurately characterizing chromosomal status, particularly in miscarriages with apparently normal karyotypes. The advent of NGS has enabled more precise chromosomal analysis; however, studies linking early ultrasonographic findings with NGS‐confirmed chromosomal status, especially focusing on euploid miscarriages, remain limited. Unlike previous reports, the present study specifically focused on NGS‐confirmed euploid early miscarriages, providing novel insight into the clinical and ultrasonographic features of early miscarriages.

The usefulness of NGS‐based chromosomal analysis of chorionic villi is clearly demonstrated by the patient selection flowchart (Figure 1). With G‐banding analysis, chromosomal results could not be obtained in 7/105 cases (6.7%) due to culture failure. In contrast, because NGS‐based analysis does not require a cell culture process, interpretable chromosomal results were successfully obtained in all cases in this study. This finding is consistent with previous reports demonstrating the high analytical success rate of NGS‐based chromosomal analysis in products of conception from miscarriage cases [14].

This study demonstrates the distinct clinical characteristics of normal and abnormal karyotype miscarriages. Normal karyotype miscarriages occurred more frequently before FHB or YS confirmation, and the mean CRL was greater in the euploid group than in the aneuploid group, whereas abnormal karyotype miscarriages tended to occur after FHB confirmation, particularly when the CRL is < 10 mm. Furthermore, normal karyotype miscarriages showed a bimodal distribution, suggesting a heterogeneous underlying mechanism. Moreover, normal karyotype miscarriages were more often associated with SCH. These findings suggest that clinical parameters, including ultrasonographic findings, may help estimate chromosomal abnormalities in early miscarriages, although their diagnostic performance is limited.

Previous studies have reported that miscarriages occurring before FHB confirmation and in the absence of YS are more frequently associated with normal karyotypes [15], which is consistent with the trend observed in our study. Two or more previous BPLs observed in the normal karyotype group may indicate that these pregnancies tend to fail at an early stage because of nonchromosomal factors such as maternal immunological, endocrinological, or uterine conditions. Because chromosomally normal conceptuses have developmental potential, implantation is more likely to occur; however, subsequent pregnancy loss may be triggered by maternal or gestational factors. Recent evidence also suggests that BPL may not primarily reflect severe embryonic chromosomal abnormalities. Kuwabara et al. [16] reported that even after the transfer of PGT‐A–confirmed euploid embryos, patients with RPL exhibited a significantly higher incidence of BPL than those with repeated assisted reproduction failure, indicating that maternal or endometrial factors may play a major role in very early pregnancy losses.

Consistent with this observation, in our study, BPL occurred more frequently in the normal karyotype group, reinforcing the interpretation that BPL is more strongly associated with maternal background factors than with embryonic chromosomal abnormalities.

Several studies have reported miscarriages following the transfer of euploid embryos in which abnormal endometrial microbiota were identified, indicating that maternal or intrauterine factors may underlie euploid losses [17, 18]. Consistently, Moreno et al. demonstrated that women who experienced clinical miscarriage after euploid embryo transfer exhibited distinctive endometrial microbial profiles compared with those with ongoing pregnancies [17]. More recently, a study showed that the endometrial microbiota composition significantly influenced pregnancy outcomes after frozen euploid embryo transfer, reinforcing the concept that intrauterine microbial imbalance may predispose women to miscarriage despite the transfer of chromosomally normal embryos [19]. In line with these reports, our present findings suggest that endometrial dysbiosis may represent a maternal risk factor contributing to euploid miscarriage, particularly in cases occurring before FHB detection. Importantly, our study extends prior work by demonstrating this association in spontaneous miscarriages rather than only in assisted reproduction. However, the relatively small number of cases is a limitation, and caution is required when interpreting and generalizing these results. Larger prospective studies are necessary to clarify the relationship between euploid miscarriages and endometrial dysbiosis.

SCH may be a characteristic feature of normal karyotype miscarriages. Previous studies have demonstrated that the presence of SCH in early pregnancy is associated with an increased risk of subsequent miscarriage [20]; however, most reports have not stratified outcomes by fetal karyotype. In our cohort, SCH occurred significantly more frequently in normal than in abnormal karyotype miscarriages. Notably, among the eight normal karyotype miscarriages with SCH, five occurred before FHB confirmation. These observations indicate that even when the embryo is chromosomally normal, maternal or intrauterine environmental factors, such as SCH, may lead to a miscarriage. Collectively, these findings support the role of SCH as a clinically meaningful maternal risk factor for normal karyotype miscarriages.

Interestingly, as noted above, normal karyotype miscarriages were more frequently observed before FHB confirmation but then showed an increase in frequency around 8 weeks of gestation, thereby demonstrating a bimodal distribution. This bimodal pattern suggests that the mechanisms underlying normal karyotype miscarriages may be heterogeneous. Very early losses before the onset of cardiac activity could reflect implantation failure or early embryonic developmental arrest, whereas losses occurring at approximately 8 weeks may be associated with maternal factors, placental dysfunction, or other non‐chromosomal etiologies. The recognition of this bimodal pattern provides novel insights into the pathophysiology of early pregnancy loss. This also emphasizes the need for further studies to delineate the biological processes specific to each peak, which may ultimately help refine both risk stratification and targeted preventive strategies.

In contrast, the lower number of previous live births may indicate a trend toward reduced subsequent live birth rates. This finding suggests that, without appropriate evaluation and management, women experiencing normal karyotype miscarriages may be at increased risk of further pregnancy loss. Accordingly, miscarriages in women with no prior live births may be considered higher risk, and a comprehensive evaluation may therefore be warranted in preparation for a subsequent pregnancy.

The possibility of selection bias should be considered when interpreting the study findings because chromosomal analysis was performed only in patients who decided to undergo testing.

This study has some limitations. First, endometrial microbiota testing was performed in miscarriage cases in which normal karyotype was confirmed by NGS analysis, in order to investigate potential causes other than fetal chromosomal abnormalities. Because chromosomal abnormalities account for a large proportion of miscarriages, evaluation of maternal factors becomes particularly relevant in cases of euploid miscarriage. Therefore, at our institution, endometrial microbiota testing is primarily performed in cases with normal karyotype. As a result, a potential selection bias may exist in the population undergoing microbiota testing, and the analysis of endometrial microbiota in this study was largely restricted to euploid miscarriage cases, limiting direct comparison with aneuploid cases. In addition, the relationship between endometrial microbiota and RPL remains unclear [21], and the available evidence is still limited. Therefore, the results of endometrial microbiota testing in the present study should be interpreted with caution. Further studies with larger and more balanced cohorts are required to clarify the clinical significance of endometrial microbiota in miscarriage.

Second, this study was the small number of patients with APS (*n* = 3). Although APS is classically 8 of 9 *Reproductive Medicine and Biology*, 2026 associated with pregnancy loss occurring after FHB confirmation, early miscarriages may also occur, particularly in patients with coexisting maternal factors. In our cohort, all APS‐positive patients experienced miscarriages before FHB confirmation, making it difficult to draw firm conclusions regarding the relationship between APS and the timing of miscarriage. Studies with larger samples are needed to clarify these clinical patterns.

Although NGS is robust against maternal cell contamination, it is inherently limited in the detection of ploidy abnormalities, including triploidy. In the present NGS platform, triploidy was evaluated in cases reported as 46,XX; however, such assessment is not fully applicable to cases reported as 46,XY, and therefore, the possibility of undetected ploidy abnormalities cannot be completely excluded. This limitation should be considered when interpreting our findings, particularly in comparison with the findings of previous studies based on G‐banding karyotype analysis.

Taken together, these findings emphasize that chromosomally abnormal miscarriages are primarily attributable to de novo genetic errors, whereas chromosomally normal miscarriages, including those presenting with BPL, are more likely to result from maternal or gestational factors. In this study, we observed that miscarriages occurring before FHB confirmation, particularly before yolk sac visualization, were more frequently associated with a normal karyotype. Therefore, chorionic villus chromosome analysis is recommended in such cases to exclude genetic causes. If genetic testing cannot be performed, clinicians should remain aware of the potential for chromosomally normal miscarriages and incorporate this into management strategies for subsequent pregnancies.

## Funding

This work was supported by the Japan Society for the Promotion of Science, 22K16887, 24K12637, 25K12643.

## Ethics Statement

The collection and use of miscarriage specimens were approved by the Ethics Committee of the Nippon Medical School Hospital (A‐2021‐058). All procedures followed were in accordance with the ethical standards of the responsible committee on human experimentation (institutional and national) and with the Helsinki Declaration of 1964 and its later amendments.

## Consent

Informed consent was obtained from all patients for being included in the study.

## Conflicts of Interest

The authors declare no conflicts of interest.

## Data Availability

The data that support the findings of this study are fully provided in the text. If further personal patient data is required, kindly make an appropriate request to the corresponding author because it cannot be posted directly in the text or made publicly available because of privacy restrictions.
